# Functional assessment of triheteromeric NMDA receptors containing a human variant associated with epilepsy

**DOI:** 10.1113/JP277292

**Published:** 2019-01-30

**Authors:** Katie F. M. Marwick, Kasper B. Hansen, Paul A. Skehel, Giles E. Hardingham, David J. A. Wyllie

**Affiliations:** ^1^ Centre for Discovery Brain Sciences Hugh Robson Building University of Edinburgh Edinburgh UK; ^2^ Department of Biomedical and Pharmaceutical Sciences Center for Structural and Functional Neuroscience and Center for Biomolecular Structure and Dynamics University of Montana Missoula MT USA; ^3^ UK Dementia Research Institute University of Edinburgh Edinburgh UK; ^4^ Centre for Brain Development and Repair Institute for Stem Cell Biology and Regenerative Medicine Bangalore India

**Keywords:** Genetics, intellectual disability, electrophysiology, Human, Epilepsy

## Abstract

**Key points:**

- NMDA receptors are neurotransmitter‐gated ion channels that are critically involved in brain cell communication
- Variations in genes encoding NMDA receptor subunits have been found in a range of neurodevelopmental disorders.
- We investigated a *de novo* genetic variant found in patients with epileptic encephalopathy that changes a residue located in the ion channel pore of the GluN2A NMDA receptor subunit.
- We found that this variant (GluN2A^N615K^) impairs physiologically important receptor properties: it markedly reduces Mg^2+^ blockade and channel conductance, even for receptors in which one GluN2A^N615K^ is co‐assembled with one wild‐type GluN2A subunit.
- Our findings are consistent with the GluN2A^N615K^ mutation being the primary cause of the severe neurodevelopmental disorder in carriers.

**Abstract:**

NMDA receptors are ionotropic calcium‐permeable glutamate receptors with a voltage‐dependence mediated by blockade by Mg^2+^. Their activation is important in signal transduction, as well as synapse formation and maintenance. Two unrelated individuals with epileptic encephalopathy carry a *de novo* variant in the gene encoding the GluN2A NMDA receptor subunit: a N615K missense variant in the M2 pore helix (*GRIN2A* ^C1845A^). We hypothesized that this variant underlies the neurodevelopmental disorders in carriers and explored its functional consequences by electrophysiological analysis in heterologous systems. We focused on GluN2A^N615K^ co‐expressed with wild‐type GluN2 subunits in physiologically relevant triheteromeric NMDA receptors containing two GluN1 and two distinct GluN2 subunits, whereas previous studies have investigated the impact of the variant in diheteromeric NMDA receptors with two GluN1 and two identical GluN2 subunits. We found that GluN2A^N615K^‐containing triheteromers showed markedly reduced Mg^2+^ blockade, with a value intermediate between GluN2A^N615K^ diheteromers and wild‐type NMDA receptors. Single‐channel conductance was reduced by four‐fold in GluN2A^N615K^ diheteromers, again with an intermediate value in GluN2A^N615K^‐containing triheteromers. Glutamate deactivation rates were unaffected. Furthermore, we expressed GluN2A^N615K^ in cultured primary mouse cortical neurons, observing a decrease in Mg^2+^ blockade and reduction in current density, confirming that the variant continues to have significant functional impact in neuronal systems. Our results demonstrate that the GluN2A^N615K^ variant has substantial effects on NMDA receptor properties fundamental to the roles of the receptor in synaptic plasticity, even when expressed alongside wild‐type subunits. This work strengthens the evidence indicating that the GluN2A^N615K^ variant underlies the disabling neurodevelopmental phenotype in carriers.

## Key points

- NMDA receptors are neurotransmitter‐gated ion channels that are critically involved in brain cell communication
- Variations in genes encoding NMDA receptor subunits have been found in a range of neurodevelopmental disorders.
- We investigated a *de novo* genetic variant found in patients with epileptic encephalopathy that changes a residue located in the ion channel pore of the GluN2A NMDA receptor subunit.
- We found that this variant (GluN2A^N615K^) impairs physiologically important receptor properties: it markedly reduces Mg^2+^ blockade and channel conductance, even for receptors in which one GluN2A^N615K^ is co‐assembled with one wild‐type GluN2A subunit.
- Our findings are consistent with the GluN2A^N615K^ mutation being the primary cause of the severe neurodevelopmental disorder in carriers.

## Introduction

The NMDA receptor is an ionotropic voltage‐dependent glutamate receptor with important roles in synaptic signal transduction and plasticity. NMDA receptor dysfunction has been identified in diverse neuropsychiatric disorders (Paoletti *et al*. 2013). Unlike AMPA‐ and kainate‐type glutamate receptors, it shows high Ca^2+^ permeability (MacDermott *et al*. 1986) and voltage‐dependent Mg^2+^ blockade, which allows it to act as a molecular coincidence detector (Mayer *et al*. 1984; Nowak *et al*. 1984). NMDA receptors are heterotetramers comprised of two GluN1 and two GluN2 subunits of which four different subunits have been cloned (GluN2A‐D) (Watanabe *et al*. 1992; Monyer *et al*. 1994). The identity of the GluN2 subunits in the receptor impacts on receptor properties important in synaptic plasticity (Wyllie *et al*. 2013). Diheteromeric GluN1/GluN2B receptors probably form the commonest forebrain receptor composition prenatally but, after GluN2A expression increases postnatally (Bar‐Shira *et al*. 2015), triheteromeric GluN1/GluN2A/GluN2B receptors comprise a major proportion of NMDA receptors, particularly in the hippocampus (Gray *et al*. 2011; Rauner & Köhr, 2011; Tovar *et al*. 2013).

The rapid expansion of whole‐exome sequencing over the last decade has shown that many regions of the gene encoding GluN2A (*GRIN2A*) are intolerant of variation in healthy controls (Ogden *et al*. 2017) and, instead, a large number of rare and *de novo* variants (>100) have been identified in patients with a range of neurodevelopmental disorders, most commonly epilepsy aphasia syndromes or other epileptic disorders, but also intellectual disability, autism, attention deficit hyperactivity disorder and schizophrenia (XiangWei *et al*. 2018). At the most severe end of the phenotypic spectrum are epileptic encephalopathies, where epileptic activity is considered to contribute to severe cognitive and behavioural impairment (Scheffer *et al*. 2017). Around half of disease‐associated *GRIN2A* variants are gene‐disrupting and around half are missense variants, potentially resulting in NMDA receptors with altered function, requiring electrophysiological interrogation for confirmation. This has been a clinically useful strategy, with personalized treatment being offered based on a functional analysis of the variant (Pierson *et al*. 2014). However, it is improbable that all variants are disease‐causing: some are inherited from phenotypically normal parents and some are in regions of the protein that probably exhibit variation without deleterious functional impact (less highly conserved, or more variation present in healthy controls). Furthermore, of those variants where functional consequences have been assessed, a range of effects have been found, some predominantly ‘gain‐of‐function’, some predominantly ‘loss‐of‐function’ (Swanger *et al*. 2016) and some with no effect (Marwick *et al*. 2017). For these reasons, it is important to identify the functional consequences, if any, of a given variant before giving a genetic diagnosis or embarking on targeted treatment.

The large and increasing number of *GRIN2A* variants (and variants in other genes) found in patients with neurodevelopmental disorders means that some prioritization for functional investigation needs to be applied. The variant that we selected for intensive further study was GluN2A^N615K^. This variant was selected because it affects a residue that is already known to be crucial in interacting with Mg^2+^ ions, the ‘N+1’ asparagine (Wollmuth *et al*. 1998). Missense mutations affecting this residue have a high probability of influencing receptor function. Second, the variant has good genetic evidence of disease‐association: it has arisen *de novo* in two unrelated individuals with similar phenotypes (Endele *et al*. 2010; Allen *et al*. 2016). Both individuals presented with early‐onset epileptic encephalopathy, associated with severe or profound intellectual disability and electroencephalogram abnormalities. Furthermore, so far, the residue has not been found to be mutated in 60 706 people without severe paediatric disease (Exome Aggregation Consortium database, accessed 3 September 2018).

The GluN2A^N615K^ variant was the first missense *GRIN2A* variant identified as potentially disease causing and some functional work on its consequences has already been performed when expressed as diheteromers: GluN2A^N615K^ has been found to reduce Mg^2+^ blockade and Ca^2+^ permeability and impact channel blocker potency but not to affect glutamate or glycine potency (Endele *et al*. 2010; Pierson *et al*. 2014). However, no previous work has investigated the impact of this variant when expressed in triheteromers with one wild‐type and one mutated GluN2 subunit. This insight would be clinically relevant, both because the variant is present heterozygously and because the majority of NMDA receptors in key regions of the adolescent and adult brain are probably GluN1/GluN2A/GluN2B triheteromers. Moreover, no previous work has investigated the impact of the variant on NMDA receptors expressed in neurons, where neuron‐specific trafficking and regulation could potentially negate or compensate for the impact of the variant.

In the present study, we employed a recently developed technique to express triheteromeric NMDA receptors containing only one variant subunit in HEK293T cells and assessed the impact of the variant on physiologically relevant receptor properties important for synaptic transmission using electrophysiological recordings. In addition, we expressed the mutant subunit in primary cultured neurons and found that the GluN2A^N615K^ mutation had a marked impact on key physiological properties of NMDA receptors even in the presence of wild‐type subunits, which is consistent with a role in the pathogenesis of the epileptic encephalopathies experienced by its carriers.

## Methods

### Ethical approval

Experiments conducted during the course of this study received approval from the University of Edinburgh's Animal Welfare Ethical Review Board. Animal breeding and maintenance and experimental procedures were performed in accordance with the UK Animals (Scientific Procedures) Act 1986 under the authority of Project Licence 60/4290 (D.J.A.W.). Animal experiments adhered to the ethical principles required by *The Journal of Physiology* (Grundy, 2015). Mice were housed under a standard 12:12 h light/dark cycle and received food and water *ad libitum*. E17.5 CD1 mice (sex not determined) supplied by Charles River (Margate, UK), were culled by decapitation (a Schedule 1 Method) shortly following culling of the dam by cervical dislocation (a Schedule 1 method). Dams were typically 12–22 weeks old.

### Mutagenesis

The cDNA for wild‐type human NMDA subunit GluN1‐1a (hereafter GluN1) and GluN2A (GenBank accession codes: NP_015566, NP_000824) (Hedegaard *et al*. 2012) were gifts from Dr Hongjie Yuan (University of Emory, Atlanta, GA, USA). The GluN2 cDNAs for triheteromer experiments have been described previously: wild‐type rat GluN2A (D13211) and GluN2B (U11419), GluN2A_C1_, GluN2A_C2_, GluN2B_AC1_ and GluN2B_AC2_ (Hansen *et al*. 2014). Expression of GluN1 in HEK293T cells was achieved as described previously (Yi *et al*. 2018) using a plasmid DNA construct with enhanced green fluorescent protein (eGFP) inserted between the cytomegalovirus promoter in pCI‐neo and the open reading frame of rat GluN1 (U08261) (i.e. eGFP and GluN1 were not expressed as a fusion protein). This DNA construct produces high expression of eGFP for identification of transfected cells and maintains a linear relationship between eGFP and GluN1 expression. All cDNAs were in pCI‐neo. Site‐directed mutagenesis was performed via PCR with overlapping mutagenizing oligonucleotides using a thermostable Pfu high fidelity DNA polymerase (New England Biolabs, Ipswich, MA, USA). The PCR product was recircularized into a viable plasmid using an InFusion HD kit (Clontech, Mountain View, CA, USA). The double‐stranded mutant DNA was transformed into TOP10 Competent Cells (Life Tech, Grand Island, NY, USA). Clones were amplified then DNA extracted using QIAPrep Spin MiniPrep Kit (Qiagen, Venlo, The Netherlands) in accordance with the manufacturer's instructions. The mutations were verified by Sanger sequencing through the mutated region.

### Preparation and transfection of HEK293T cells

For whole‐cell experiments, human embryonic kidney 293 cells containing the SV40 large T–antigen (HEK293T) cells were cultured in Dulbecco's modified Eagle's medium supplemented with 10% dialysed fetal bovine serum, 10 U mL^–1^ penicillin and 10 mg mL^–1^ streptomycin). HEK293T cells were chosen for their propensity to grow singly rather than in clumps, facilitating electrophysiological experiments involving rapid solution application. Cells were passaged twice weekly and plated onto cover slips precoated in poly‐d‐lysine (0.1 mg mL^–1^) to give a density of 10–30% on the day of recording. HEK293T cells were transfected using the calcium phosphate precipitation method. Plasmids containing rGluN1‐1a eGFP and the rGluN2 subunits of interest were mixed in a 1:1 mass ratio and diluted to 200 g L^–1^ with water. To transfect four wells of a 24‐well plate, 10 μL of cDNA was mixed with 25 μL of 1 m CaCl_2_ and 100 μL of 2 × Bes (50 mm Bes, 280 mm NaCl and 1.5 mm Na_2_HPO_4_, pH 6.95), then mixed by pipetting. After 10–15 min, 50 μL of this mixture was added dropwise to each well. After 4–6 h, the media was replaced with fresh media supplemented with NMDA receptor antagonists [d‐2‐amino‐5‐phosphonopentanoic acid (200 μm) and 7‐chlorokynurenic acid (200 μm)]. Recordings were made ∼24 h post transfection. HEK293T for single‐channel experiments were prepared similarly with minor differences: Dulbecco's modified Eagle's medium was supplemented with Glutamax‐I (Thermo Fisher, Waltham, MA, USA) and 1% antibiotic/antimycotic and the NMDA receptor antagonists used to avoid excitotoxicity post transfection were 100 μm d‐2‐amino‐5‐phosphonopentanoic acid and 10 μm 5,7‐dichlorokynurenic acid.

### Whole‐cell, voltage clamp recordings in HEK293T cells

Transfected HEK293T cells were identified by eGFP expression (excitation at 470 nm, coolLED pE‐100; coolLED Ltd, Andover, UK). Whole‐cell, patch clamp recordings were performed using an Axopatch 200B amplifier (Molecular Devices, Sunnydale, CA, USA) at room temperature. The signal was then filtered using an 8 kHz 8‐pole low‐pass filter (–3 dB Bessel; Frequency Devices, Ottawa, IL, USA) and digitized at 20 kHz using a Digidata 1440A analogue‐digital interface (Molecular Devices) using Clampex software (Molecular Devices). Patch pipettes were made from thin‐walled borosilicate glass (TW150F‐4; World Precision Instruments, Sarasota, FL, USA) using a P‐1000 puller (Sutter Instruments, Novato, CA, USA) to give a resistance of 4–5 MΩ when filled with an internal solution containing (in mm): 141 K‐gluconate, 2.5 NaCl, 10 Hepes and 11 EGTA (pH 7.3 with KOH) (300 mosmol L^−1^). The extracellular solution was composed of (in mm): 150 NaCl, 10 Hepes, 3 KCl, 0.5 CaCl_2_, 0.01 EDTA and 20 d‐mannitol (pH 7.4 with NaOH). Rapid solution exchange (open tip solution exchange with 10–90% rise times of 0.7–0.9 ms) was achieved using a two‐barrel theta‐glass pipette controlled by a piezoelectric translator (Siskiyou Corporation, Grants Pass, OR, USA). Cells with glutamate‐evoked responses of less than 100 pA or greater than 1000 pA were rejected, to reduce error from noise and to avoid leak of undesired subunit pairings (Hansen *et al*. 2014). To calculate glutamate deactivation rates, multicomponent exponential decay curves were fitted to macroscopic response time courses in ChanneLab software (Synaptosoft, Fort Lee, NJ, USA).

t weighted  was  calculated  as :% amplitud e fast ∗t fast +(% amplitud e slow ∗t slow )

### Single‐channel, voltage clamp recordings in HEK293T cells

Single‐channel, voltage clamp recordings were made at room temperature from cell‐attached patches formed on HEK293T cells placed in a solution that contained (in mm): 150 NaCl, 2.8 KCl, 10 Hepes, 2 CaCl_2_, 10 glucose and 0.1 glycine (pH 7.35 with NaOH) (300–330 mosmol L^−1^). Patches were recorded at a range of holding potentials. Transfected cells were identified by eGFP expression as above. Patch pipettes were made from thick‐walled borosilicate glass (GC150F‐7.5; Harvard Apparatus, Cambridge, MA, USA) using a P‐87 puller (Sutter Instruments, Novato, CA, USA) and fire‐polished to give a resistance of 6–12 MΩ when filled with the external solution plus agonist (glutamate 1 mm). Currents were recorded using an Axopatch 200B amplifier (Molecular Devices). Data were filtered at 2 kHz and digitized at 20 kHz via a BNC‐2090A analogue‐digital interface (National Instruments, Newbury, UK) using WinEDR software (Strathclyde Electrophysiology Software, Strathclyde, UK).

WinEDR v3 (Strathclyde Electrophysiology Software) was used to idealize the traces (using a transition threshold of 50% of the predominant conductance level and a 100 μs open and shut resolution) and to fit Gaussian curves to amplitude histograms. The relative proportion of time spent in a given conductance state was calculated by fitting areas under the amplitude histograms. To improve accuracy of amplitude estimates, a 1 ms minimum open time duration was applied. Because the membrane potential was not known, the patch potential was unknown. Therefore, recordings were made at a range of pipette potentials (+40 to +180 mV) and the current plotted against voltage: the slope (fitted by linear regression) gave the conductance. Mean open times were calculated using openings at one pipette potential, with a 1 ms minimum open time duration applied, and fitted using single exponentials in WinEDR. Transitions between subconductance states were manually coded from inspection of all openings with duration >1 ms at a single pipette potential. Traces were excluded if: *R* ^2^ < 0.9 for the linear regression, fewer than three pipette potentials were recorded from, or fewer than 20 openings occurred at a given pipette potential.

### Preparation and transfection of neurons

Neuronal culturing and transfection was performed as described previously (Marwick *et al*. 2017). Shortly following decapitation, brains were microdissected in medium containing (in mm): 8.8 Na_2_SO_4_, 27 K_2_SO_4_, 5.3 MgCl_2_, 0.23 CaCl_2_, 0.9 Hepes, 0.001% phenol red, 18 d‐glucose and 0.0005 kynurenic acid (adjusted to pH 7.35 using NaOH). Cortices were incubated for 40 min at 37°C in papain enzyme (36,000 USP units mL^–1^) (Worthington Biochemical Corporation, Lakewood, NJ, USA) then washed and triturated in NeuroBasal A medium (Thermo Fisher) (supplemented with 1% rat serum; Harlan Laboratories, Indianapolis, IN, USA), 1 × B‐27 supplement, 1% antibacterial/antimycotic and 1 mm glutamine). Opti‐MEM (supplemented with 20 mm glucose and 1% antibacterial/antimycotic) was added to the cell suspension to give an end concentration of 1 hemisphere per 7 mL, and 0.5 mL was plated onto a glass coverslip (diameter 13 mm) precoated with poly‐d‐lysine (1.33% w/v in H_2_O) and laminin (0.5% w/v) (Roche, Basel, Switzerland) in 24‐well plates. Plates were kept for 2.5 h in a humidified 5% CO_2_ incubator at 37°C before the cell suspension was removed and replaced with supplemented NeuroBasal A. On days *in vitro* (DIV) 4, 1 mL well^–1^ of supplemented NeuroBasal A additionally containing 9.6 mm cytosine β‐d‐arabinofuranoside hydrochloride (a DNA replication inhibitor that prevents glial overproliferation) was added to the cells.

Neurons were transfected on DIV 7 or 8 with plasmids containing cDNA for wild‐type and mutant GluN2A subunits, or the inert control β globin, using Lipofectamine 2000 (Thermo Fisher) (hereafter referred to as lipofectamine) in serum free non‐trophic transfection medium composed of: 10% minimum essential media (+Earles, –l‐glutamine), 88% salt‐glucose‐glycine (comprising in mm 114 NaCl, 26 NaHCO_3_, 5.3 KCl, 1 MgCl_2_, 2 CaCl_2_, 10 Hepes, 1 glycine, 30 d‐glucose and 0.5 sodium pyruvate, with phenol red 0.001%) (Bading *et al*. 1993) supplemented with 1% antibiotic/antimycotic and 1% insulin–transferrin–selenium supplement. For each coverslip, 575 ng of plasmid DNA comprising a 2:1 mass ratio of GluN2A/GluN2B and eGFP cDNA was mixed with 2.3 μL of lipofectamine. The cotransfection rate was 96% (data not shown). Electrophysiological recordings were performed ∼48 h post transfection.

### Whole‐cell, voltage clamp recordings in cultured neurons

Electrophysiological recordings in cultured neurons were performed as described previously (Marwick *et al*. 2017). Recordings were made at room temperature with neurons superfused (flow rate of 2 mL min^–1^) with external recording solution composed of (in mm) 150 NaCl, 2.8 KCl, 10 Hepes, 2 CaCl_2_, 10 glucose, 0.1 glycine and 0.003 TTX (pH 7.35 using NaOH) (300–330 mosmol L^−1^). Transfected cells were identified using eGFP expression (excitation at 470 nm, coolLED pE‐100; coolLED Ltd). Then, 150 μm NMDA was applied briefly twice to trigger desensitization until a steady response was achieved (∼10 s) at which point 1 mm MgCl_2_ was co‐applied until the response plateaued. Cells were then perfused with 3 μm ifenprodil for 1 min before the experiments were repeated. Solution application was manually controlled. Patch pipettes were made from thick‐walled borosilicate glass (GC150F‐7.5; Harvard Apparatus) using a P‐87 puller (Sutter Instruments) to give a resistance of 2–4 MΩ when filled with internal solution containing (in mm): 141 K‐gluconate, 2.5 NaCl, 10 Hepes and 11 EGTA (pH 7.3 with KOH) (300–330 mosmol L^−1^). Currents were recorded using an Axopatch 200B amplifier (Molecular Devices). Data were filtered at 2 kHz and digitized at 20 kHz via a National Instruments BNC‐2090A analogue–digital interface (National Instruments) using WinEDR software. Neurons were voltage clamped at –65 mV, and recordings were rejected if the holding current was greater than 150 pA or if the series resistance was greater than 30 MΩ, or increased by greater than 20% during the course of the recording. Capacitance was calculated by calculating the area under the current response to a 5 mV test pulse plotted against time (giving the charge) and dividing by the voltage of the test pulse. Current density was then calculated as current/capacitance.

### Statistical analysis

Data are presented as the mean ± SEM. Graphs depict individual cells (circles), means (columns) and SEM (error bars). *N* refers to number of cells. R, version 3.1.2 (R Core Team, 2014) was used to perform statistical tests. Comparisons between multiple means are performed by ANOVA, with *post hoc* tests performed if the *F* test was significant for a main effect. Comparisons between two means are performed by independent, two‐tailed, Welch *t* tests (which do not assume equal variance between groups) unless otherwise stated. Correction for multiple comparisons is made using the Bonferroni method. *P* < 0.05 was considered statistically significant (^*^ *P* < 0.05, ^**^ *P* < 0.01 and ^***^ *P* < 0.001.

### Materials

NMDA, NMDA receptor antagonists, ifenprodil and TTX were purchased from Tocris Bioscience (Bristol, UK). Media, media supplements, Lipofectamine 2000, fetal bovine serum, antibiotic/antimycotic and Β‐27 were purchased from Invitrogen (Carlsbad, CA, USA). The remaining substances were purchased from Sigma‐Aldrich (St Louis, MO, USA) unless otherwise stated.

## Results

### GluN2A^N615K^ reduces Mg^2+^ blockade in triheteromeric NMDA receptors

**Figure 1:**
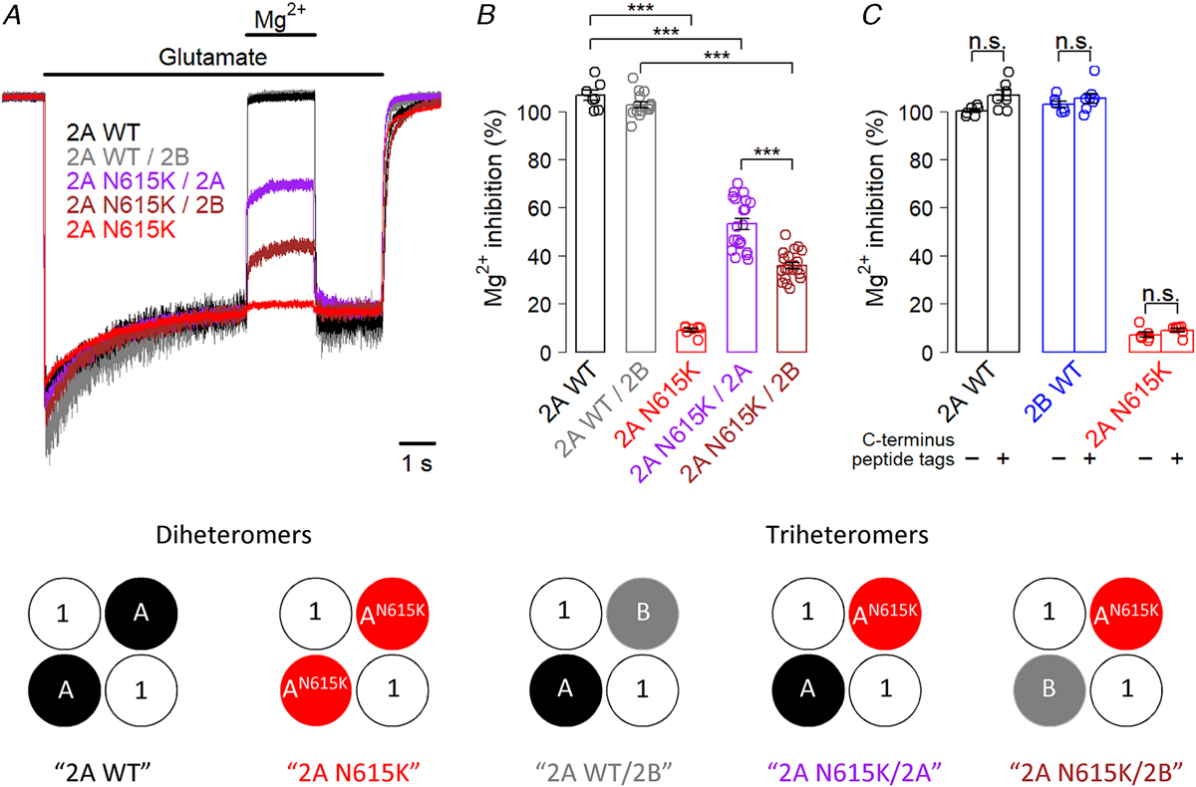
**GluN2A^N615K^ reduces Mg^2+^ blockade in triheteromeric NMDA receptors** *A*, representative whole‐cell, voltage clamp recordings from HEK293T cells expressing NMDA receptors containing wild‐type GluN2A diheteromers, GluN2A/2B triheteromers and GluN2A^N615K^‐containing diheteromers and triheteromers, partnered with either GluN2A^WT^ or GluN2B^WT^. All subunits, including wild‐type, had modified endoplasmic reticulum signals. The traces show responses to glutamate (1 mm) and blockade by Mg^2+^ (1 mm) in the continuous presence of glycine (100 μm), held at –60 mV. *B*, summary data showing percentage blockade by Mg^2+^ for each receptor composition. A one‐way ANOVA showed a significant effect of receptor subunit composition (*F*_4,64_ = 346, *P* < 2 × 10^–16^). *Post hoc* Bonferroni corrected *t* tests showed that GluN2A^N615K^ diheteromeric receptors had a significantly reduced Mg^2+^ blockade (9 ± 1%, *n* = 7) compared to GluN2A^WT^ diheteromers (107 ± 2%, *n* = 7, *t*_7.3_ = 42, *P* = 5 × 10^–10^). Triheteromeric receptors containing only one GluN2A^N615K^ subunit also showed a marked reduction in Mg^2+^ blockade, whether partnered with one GluN2A^WT^ subunit (53 ± 2%, *n* = 20), compared with GluN2A^WT^ diheteromers (*t*_19.9_ = 16.5, *P* = 2 × 10^–12^) or partnered with one GluN2B^WT^ subunit (36 ± 1%, *n* = 20), compared with GluN2A^WT^/2B^WT^ (103 ± 1%, *n* = 15, *t*_32.6_ = 36.9, *P* = 9 × 10^–16^). The reduction in Mg^2+^ blockade was greater in GluN2A^N615K^ subunits partnered with GluN2B^WT^ than with GluN2A^WT^ (*t*_29.4_ = 6.3, *P* = 3 × 10^–6^). Some traces show greater than 100% blockade by Mg^2+^, reflecting a small contamination of the bath fluid (baseline exposure) by glutamate. *C*, summary data showing percentage blockade by Mg^2+^ for NMDA receptors with and without modified C‐termini. A two‐way ANOVA showed a significant influence of C‐terminus tag (*F*_1,35_ = 8.0, *P* = 0.00785) and a significant effect of receptor subunit composition (*F*_2,35_ = 2635, *P* < 2 × 10^–16^), although there was no significant interaction (*F*_2,35_ = 1.3, *P* = 0.28). However, *post hoc* Bonferroni corrected *t* tests showed no significant effect of modifying C‐termini with engineered tags for any subunit.

We first investigated Mg^2+^ blockade in GluN1/GluN2A^N615K^ diheteromers expressed in HEK293T cells (Fig. 1). Consistent with previous work (Endele *et al*. 2010), we found that the GluN2A^N615K^ variant resulted in negligible Mg^2+^ blockade at approximately physiological concentrations. We next investigated the impact of the GluN2A^N615K^ variant when expressed as part of triheteromeric NMDA receptors containing wild‐type GluN2 subunits (Fig. 1). Accordingly, we employed receptor subunits with modified endoplasmic reticulum retention signals as developed by Hansen *et al*. (2014) to express receptors containing zero, one or two GluN2A^N615K^ subunits partnered with wild‐type GluN2A or GluN2B subunits. Receptors containing only one GluN2A^N615K^ subunit continued to exhibit markedly reduced Mg^2+^ blockade, with a blockade intermediate between wild‐type and GluN2A^N615K^ diheteromers (Fig. 1 *A* and *B*). Unexpectedly, the identity of the wild‐type subunit partnered with GluN2A^N615K^ impacted on Mg^2+^ blockade, with a GluN2B^WT^ partner showing lower Mg^2+^ blockade than a GluN2A^WT^ partner (Fig. 1 *A* and *B*). We confirmed that the modified C‐termini did not themselves influence Mg^2+^ blockade (Fig. 1 *C*).

### GluN2A^N615K^ does not influence glutamate deactivation

**Figure 2:**
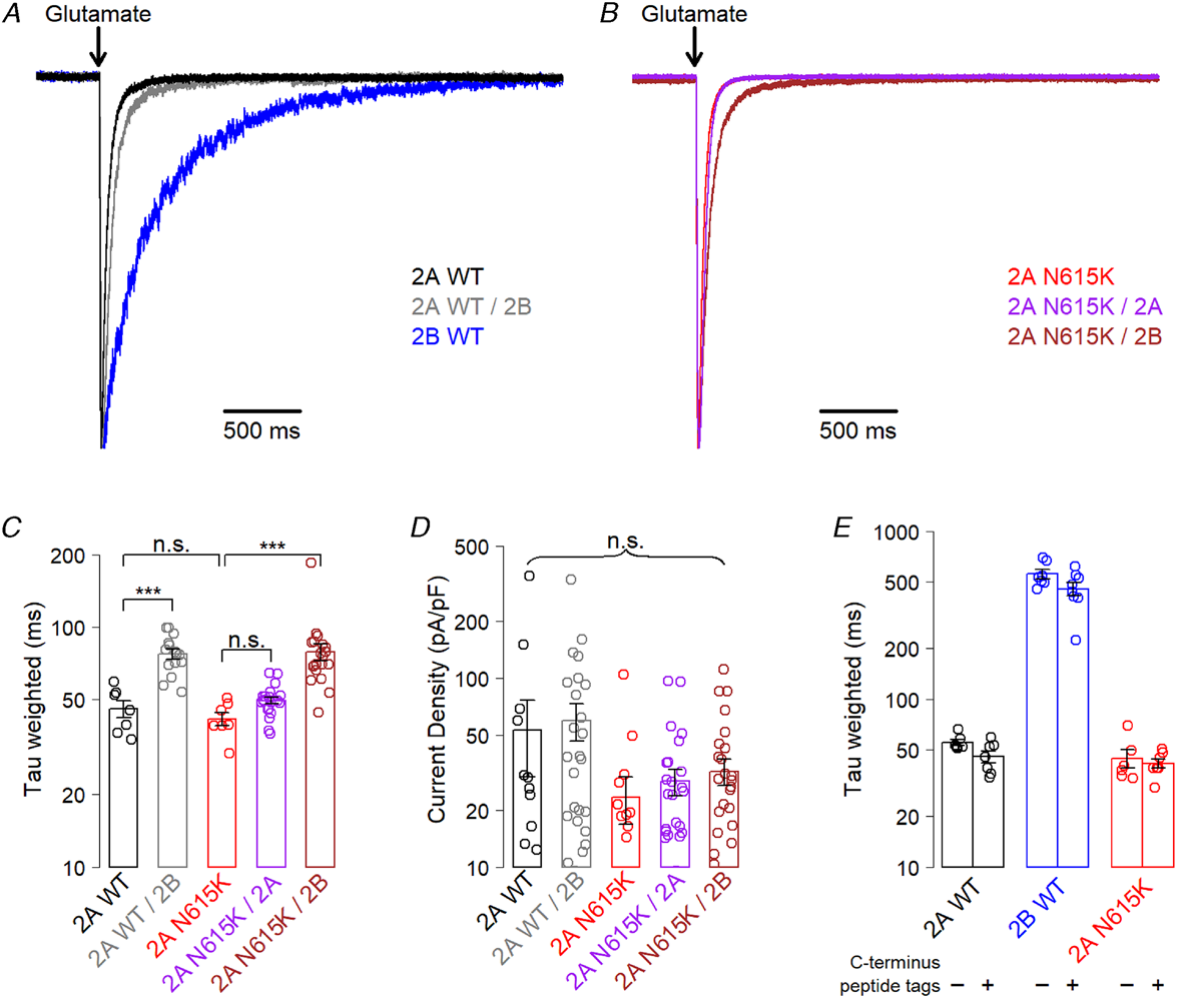
**GluN2A^N615K^ does not alter glutamate deactivation** *A* and *B*, representative whole‐cell, voltage clamp recordings from HEK293T cells expressing NMDA receptors containing wild‐type GluN2A diheteromers, GluN2A/2B triheteromers and GluN2A^N615K^ containing diheteromers and triheteromers, partnered with either GluN2A^WT^ or GluN2B^WT^. All subunits, including wild‐type, had modified endoplasmic reticulum signals. The traces show deactivation following 5 ms exposure to glutamate (1 mm) and glycine (100 μm) in the absence of Mg^2+^ when held at –60 mV. Responses are normalized to their peak amplitudes. *C*, summary data showing weighted time constants fitted to the glutamate deactivation curves. A one‐way ANOVA showed a significant effect of receptor subunit composition (*F*_4,64_ = 14, *P* = 3 × 10^–8^). *Post hoc* Bonferroni corrected *t* tests showed that GluN2A^WT^/2B triheteromers had a slower deactivation time constant (77 ± 4 ms; *n* = 15), than GluN2A^WT^ diheteromers (45 ± 4 ms; *n* = 7; *t*_17_ = 6.2, *P* = 4 × 10^–5^). GluN2A^WT^ diheteromers were indistinguishable from GluN2A^N615K^ diheteromers (41 ± 3; *n* = 7; *t*_11_ = 0.9, *P* ≥ 0.4), with GluN2A^N615K^ and GluN2A^N615K^/2A (49 ± 2 ms, *n* = 20) also showing no difference (*t*_10.9_ = 2.6, *P* = 0.1). GluN2A^N615K^/2B triheteromers (79 ± 6, *n* = 20) showed a slower deactivation rate than GluN2A^N615K^ diheteromers (*t*_10.9_ = 2.6, *P* = 5 × 10^–5^)). *D*, summary data showing current density (peak amplitude/capacitance) for cells expressing diheteromeric and triheteromeric receptor combinations (for other experiments, only cells with a current response of between 100 and 1000 pA were used to allow accuracy without escape of unwanted subunit combinations but, here, all traces are included). A one‐way ANOVA showed no effect of receptor subunit composition (*F*_4,104_ = 2.1, *P* = 0.08). *E*, summary data showing weighted time constants fitted to glutamate deactivation curves. Modifying the C‐terminus with engineered peptide tags designed to vary retention in the endoplasmic reticulum had no effect on glutamate deactivation in diheteromeric wild‐type receptors containing GluN2A^WT^ or GluN2B^WT^, or in those containing GluN2A^N615K^. A two‐way ANOVA showed no significant influence of C terminus tag (*F*_1,35_ = 4.0, *P* = 0.054), a significant effect of NMDAR subunit composition (*F*_2,35_ = 226, *P* < 2 × 10^–16^) and no significant interaction (*F*_2,35_ = 2.5, *P* = 0.1).

Previously, GluN2A/2B triheteromer glutamate deactivation rates have been found to be intermediate between those of GluN2A and GluN2B diheteromers (Hansen *et al*. 2014; Stroebel *et al*. 2014). We used fast applications of glutamate onto HEK293T cells to replicate this finding, and we also demonstrated that GluN2A^N615K^ does not influence glutamate deactivation rates (Fig. 2 *A*–*C*). Current density showed a trend towards lower levels in cells expressing GluN2A^N615K^‐containing receptors, although this was not statistically significant (Fig. 2 *D*). We also confirmed that the C‐terminus modifications required for the generation of triheteromers do not influence glutamate deactivation rates in wild‐type or GluN2A^N615K^‐containing receptors (Fig. 2 *E*).

### GluN2A^N615K^ reduces single‐channel conductance in diheteromeric and triheteromeric NMDA receptors

**Figure 3:**
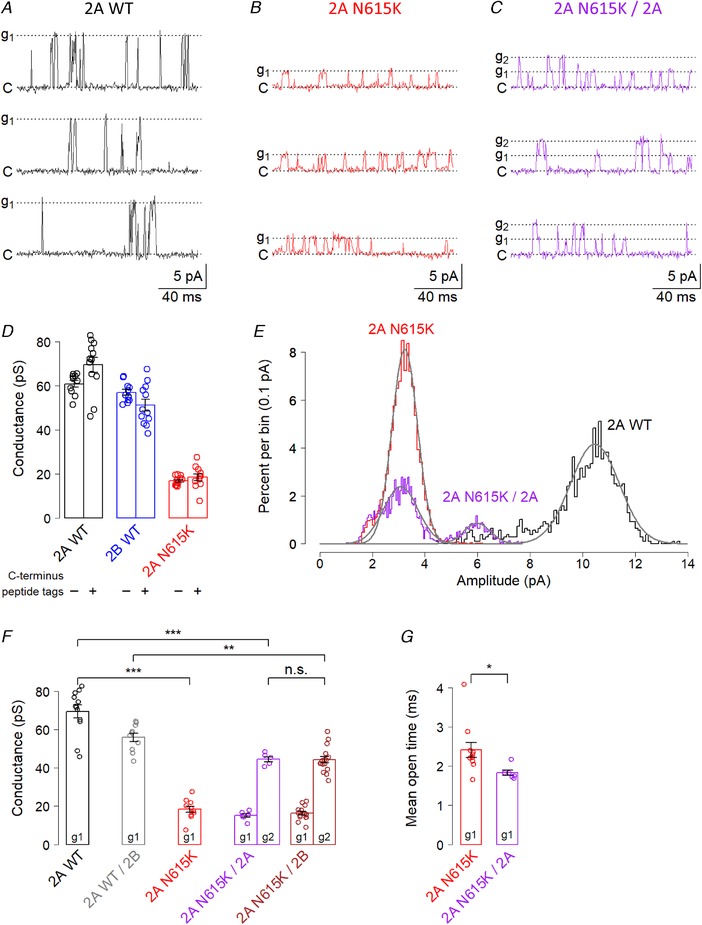
**GluN2A^N615K^ reduces single‐channel conductance in triheteromeric NMDA receptors** *A*–*C*, representative voltage clamp recordings made from cell‐attached patches from HEK293T cells expressing NMDA receptors containing wild‐type GluN2A diheteromers, GluN2A/2B triheteromers and GluN2A^N615K^‐containing diheteromers and triheteromers, partnered with either GluN2A^WT^ or GluN2B^WT^. All subunits, including wild‐type, had modified endoplasmic reticulum signals. The traces show single‐channel currents in the presence of glutamate (1 mm) and glycine (100 μm). The pipette potentials used for the traces illustrated in (*A*) to (*C*) were +120, +140 and +140 mV, respectively. ‘C’ = closed; ‘g1’ = first conductance fitted; ‘g2’ = second conductance fitted. A number of transitions between the two conductance states can be seen in (*C*). *D*, summary data showing single channel conductance for receptors with and without modified C‐termini. A two‐way ANOVA showed no significant influence of C‐terminus tag (*F*_1,62_ = 0.6, *P* = 0.42), a significant effect of receptor subunit composition (*F*_2,62_ = 278, *P* < 2 × 10^–16^) and a significant interaction (*F*_2,62_ = 5.7, *P* = 0.006). *E*, representative amplitude histograms showing fitted normal distributions, superimposed from three different patches. The means of the fitted distributions were used to calculate conductance. For the patch expressing a GluN2A^N615K^‐containing triheteromer, two peaks can be seen: the prominent subconductance and the less frequent intermediate conductance. *F*, summary data showing conductances, calculated by plotting current amplitudes against pipette potential (at least three potentials within range + 40 to + 180 mV). A two‐way ANOVA showed a main effect of subunit (*F*_4,70_ = 121, *P* < 2 × 10^–16^) and of conductance level (g1 *vs*. g2; *F*_1,70_ = 196, *P* < 2 × 10^–16^), with no significant interaction. *Post hoc* Bonferroni corrected *t* tests showed that GluN2A^N615K^ diheteromeric receptors had a significantly lower conductance (18 ± 2 pS; *n* = 11) than GluN2A^WT^ diheteromers (69 ± 3 pS; *n* = 12; *t*_15.4_ = 13.7, *P* = 2 × 10^–9^). Triheteromeric receptors containing only one GluN2A^N615K^ subunit also showed a marked reduction in intermediate conductance (g2), whether partnered with one GluN2A^WT^ subunit (44 ± 1 pS; *n* = 6), compared with GluN2A^WT^ diheteromers (*t*_13.5_ = 7.0, *P* = 3 × 10^–5^) or with one GluN2B^WT^ subunit (44 ± 1 pS; *n* = 16), compared with GluN2A^WT^/2B^WT^ (56 ± 2 pS; *n* = 10; *t*_18.4_ = 4.2, *P* = 0.003). The reduction in conductance was no greater in GluN2A^N615K^ subunits partnered with GluN2B^WT^ than with GluN2A^WT^ (*t*_19.1_ = 0.06, *P* > 0.9). *G*, summary data showing receptor mean open times calculated by fitting single exponentials to open time distributions from individual patches. Only the lower conductance openings were fitted for GluN2A^N615K^/2A triheteromers, which showed briefer open times than GluN2A^N615K^ diheteromers (2A N615K/2A: 1.83 ± 0.07 ms; *n* = 6 *vs*. 2A N615K 2.42 ± 0.19 ms, *n* = 11; *t*_12.5_ = 2.9, *P* = 0.014).

The ‘N+1’ residue altered by the GluN2A^N615K^ variant is an ion‐channel lining residue and contributes to form one of the narrowest regions of the receptor pore (Song *et al*. 2018). We therefore hypothesized that the variant would influence ion permeation in addition to Mg^2+^ blockade, and investigated single channel conductance. We found that the GluN2A^N615K^ variant lead to a substantial four‐fold reduction in single‐channel conductance in GluN2A^N615K^‐containing diheteromers (Fig. 3 *A*, *B*, *E* and *F*). As a control, we confirmed that the modified C‐termini did not influence conductance (Fig. 3 *D*).

In view of the marked effect of only one GluN2A^N615K^ subunit on Mg^2+^ blockade in NMDA receptors, we hypothesized that GluN2A^N615K^ containing triheteromeric receptors would also show a conductance intermediate between GluN2A^WT^ and GluN2A^N615K^ diheteromers. Our recordings from cells expressing triheteromeric receptors showed a more complex picture than expected: the vast majority of GluN2A^N615K^ triheteromeric patches showed channels with two conductances (Fig. 3 *C*, *E* and *F*). One conductance was indeed intermediate between that of GluN2A^WT^ and GluN2A^N615K^ diheteromers; the other was a prominent subconductance close in value to the primary conductance of GluN2A^N615K^ diheteromers.

We conducted some additional analyses to investigate two alternative explanations that could mediate what appears to be a prominent subconductance in GluN2A^N615K^ containing triheteromeric NMDA receptors. One alternative explanation is that the higher conductance represents simultaneous openings of two triheteromeric channels. However, the higher conductance was equal to more than double the lower conductance (44 pS *vs*. 15 pS and 44 pS *vs*. 16 pS) (Fig. 3 *F*). We therefore concluded that the upper conductance did not represent the simultaneous opening of two identical lower conductance triheteromeric channels.

Another alternative explanation could be that the patch contains one triheteromeric channel and one diheteromeric channel that has ‘escaped’ retention in the endoplasmic reticulum. To explore this possibility, we compared events attributable to the simultaneous opening and/or closing of two channels in patches expressing triheteromers *vs*. patches expressing multiple diheteromeric GluN2A^N615K^ receptors and found far fewer such events associated with multiple diheteromers than with triheteromers [GluN2A^N615K^ diheteromers: 4 ± 2% of openings (*n* = 7) *vs*. GluN2A^N615K^/2A triheteromers: 25 ± 1% of openings (*n* = 6) (*t*_10.1_ = 7.8, *P* = 2.8 × 10^–5^) and GluN2A^N615K^/2B triheteromers: 19 ± 2% of openings (*n* = 16) (*t*_17.0_ = 4.7, *P* = 3.9 × 10^–4^)].

We also assessed mean open times, finding that the GluN2A^N615K^/2A triheteromeric subconductance openings were briefer than those observed for GluN2A^N615K^ diheteromers (Fig. 3 *G*). This argues against the possibility that the similar conductance is mediated by GluN2A^N615K^ diheteromeric receptors present in triheteromer‐expressing patches. Such diheteromeric ‘escape currents’ can potentially arise when expression levels are high (Hansen *et al*. 2014) and, indeed, we did observe a small number of putative triheteromeric receptor patches (five out of 27 patches) in which only one conductance was observed (four with conductances close to wild‐type diheteromers; one with a conductance close to 2A N615K diheteromers). Because these patches presumably contained escaped diheteromeric receptors, they were excluded from the analysis. Overall, our findings suggest that the GluN2A^N615K^ variant results in a marked reduction in NMDA receptor single channel conductance when one or two copies are present. When two copies are present in a receptor, a single low conductance is seen. When one copy is present, two conductances are seen: low and intermediate.

### GluN2A^N615K^ reduces Mg^2+^ blockade and current density in cultured neurons

**Figure 4:**
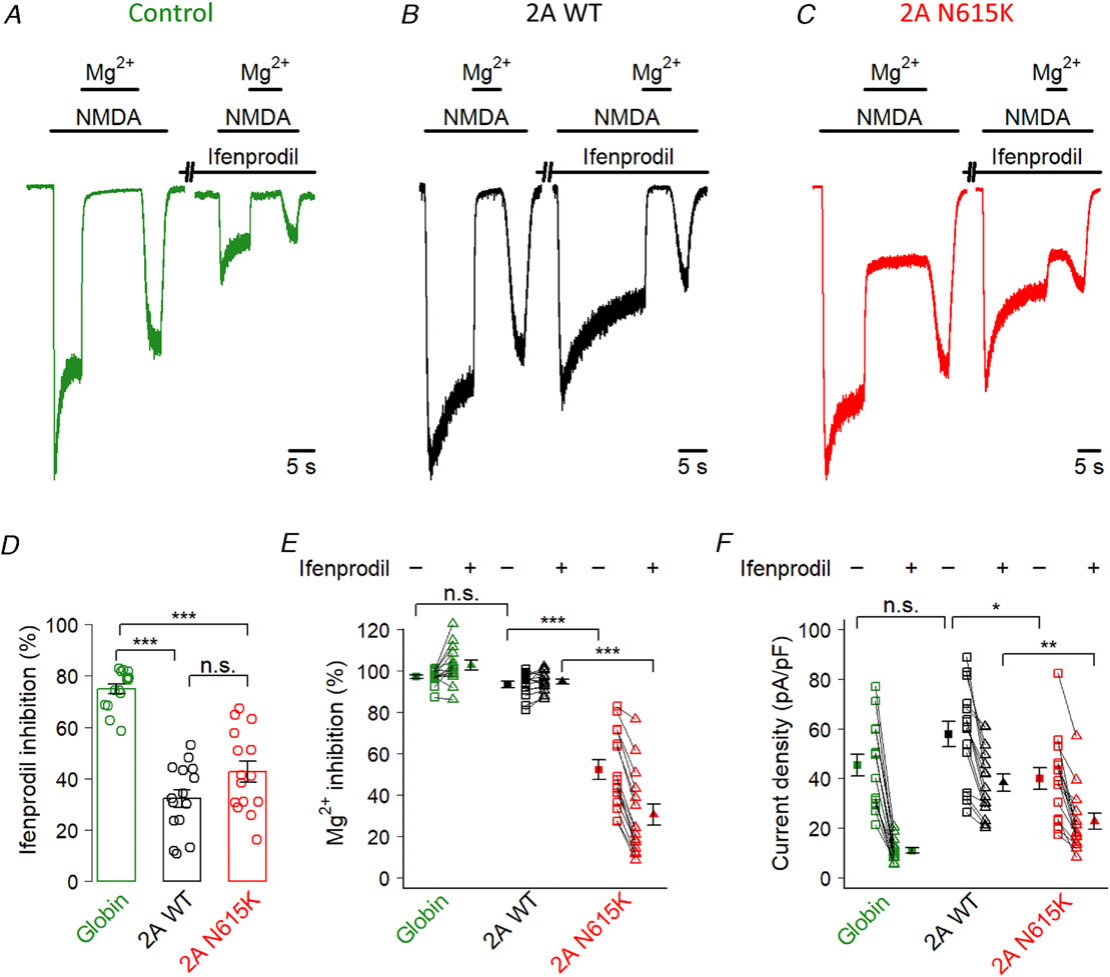
**GluN2A^N615K^ reduces Mg^2+^ blockade and current density in cultured neurons** *A*–*C*, representative whole‐cell, voltage clamp recordings made from DIV 9 primary mouse cortical neurons transiently transfected with an inert control: β globin (*A*), GluN2A^WT^ (*B*) or GluN2A^N615K^ (*C*). Traces show the response to saturating NMDA (150 μm) and inhibition by Mg^2+^ (1 mm), before and after a 1 min application of ifenprodil (3 μm) (a selective GluN2B negative allosteric modulator). *D*, summary data showing percentage inhibition by ifenprodil. A one‐way ANOVA showed a significant effect of transfected subunit (*F*_2,42_ = 46, *P* = 2.8 × 10^–11^), with *post hoc* Bonferroni corrected *t* tests indicating that neurons transfected with GluN2A^WT^ showed lower ifenprodil sensitivity than control transfection cells (WT: 32 ± 3; *n* = 15; globin 75 ± 2; *n* = 15; *t*_21.9_ = 10.7, *P* = 1.0 × 10^–9^), as did neurons transfected with GluN2A^N615K^ (43 ± 4; *n* = 15; *t*_20.0_ = 7.2, *P* = 1.9 × 10^–6^) and with no difference between GluN2A and GluN2A^N615K^ (*t*_27.3_ = 1.9, *P* = 0.19). These results confirm that the GluN2A^N615K^ subunits have been successfully trafficked to the surface in neurons. *E*, summary data showing Mg^2+^ blockade in the presence and absence of ifenprodil. Squares show blockade without ifenprodil; triangles show blockade with ifenprodil. Solid shapes represent means; hollow shapes are individual cells. A two‐way ANOVA showed a significant effect of transfected subunit (*F*_2,42_ = 111, *P* = 2 × 10^–16^) and the presence of ifenprodil (*F*_1,42_ = 31, *P* = 1.9 × 10^–6^), with a significant interaction (*F*_2,42_ = 90, *P* = 6.7 × 10^–16^). *Post hoc* Bonferroni corrected *t* tests showed lower Mg^2+^ blockade in the absence of ifenprodil in neurons transfected with GluN2A^N615K^ compared to GluN2A^WT^ (WT: 93 ± 1%; *n* = 15; N615K 52 ± 5%; *n* = 15; *t*_16.7_ = 8.2, *P* = 9.7 × 10^–7^), although there was no difference between GluN2A^WT^ and control (globin: 97 ± 1%; *n* = 15; *t*_23.3_ = 2.2, *P* = 0.13). In the presence of ifenprodil, the reduction in Mg^2+^ blockade associated with GluN2A^N615K^ *vs*. wild‐type was even more marked (WT: 95 ± 1%; *n* = 15; N615K 31 ± 5%; *n* = 15; *t*_15.4_ = 12.1, *P* = 8.4 × 10^–9^). *F*, summary data showing current density in the presence and absence of ifenprodil. As with Mg^2+^ blockade, we found that current density was reduced in neurons transfected with GluN2A^N615K^, and this effect was more pronounced in the presence of ifenprodil. A two‐way ANOVA showed a significant effect of transfected subunit (*F*_2,42_ = 9.4, *P* = 0.0004) and the presence of ifenprodil (*F*_1,42_ = 178, *P* = 2 × 10^–16^), with a significant interaction (*F*_2,42_ = 9.2, *P* = 0.0005). *Post hoc* Bonferroni corrected *t* tests showed lower current density in the absence of ifenprodil in neurons transfected with GluN2A^N615K^ compared to GluN2A^WT^ (WT: 58 ± 5 pA pF^–1^; *n* = 15; N615K 40 ± 4 pA pF^–1^; *n* = 15; *t*_27.3_ = 2.7, *P* = 0.037), although there was no difference between GluN2A^WT^ and control (globin: 45 ± 4 pA pF^–1^; *n* = 15; *t*_27.4_ = 1.9, *P* = 0.22). In the presence of ifenprodil, the reduction in current density associated with GluN2A^N615K^ *vs*. wild‐type was even more marked (WT: 38 ± 4 pA pF^–1^; *n* = 15; N615K 23 ± 3 pA pF^–1^; *n* = 15) (*t*_27.8_ = 3.3, *P* = 0.009).

To complete our investigation, we wished to explore whether the pronounced effects of GluN2A^N615K^ seen in NMDA receptors expressed in a human cell line would also be seen in receptors expressed in primary neurons, subject to neuronal specific trafficking and regulation. Accordingly, we used transient transfection to overexpress GluN2A^WT^ and GluN2A^N615K^ subunits in mouse primary cortical neurons. The resulting NMDA receptor population probably comprised a mixture of diheteromeric and triheteromeric GluN2B and GluN2A‐containing receptors, formed from both endogenous GluN2B subunits and transfected subunits. We first confirmed the expression of transfected GluN2A subunits (wild‐type or GluN2A^N615K^) by demonstrating a reduction in inhibition by ifenprodil (a selective GluN2B negative allosteric modulator) compared to neurons transfected with an inert control, which express predominantly GluN2B at DIV 9 (McKay *et al*. 2012) (Fig. 4 *A*–*D*). We next assessed the impact of the GluN2A^N615K^ mutation on Mg^2+^ blockade, in the presence and absence of ifenprodil (Fig. 4 *A*–*C* and *E*). We found that Mg^2+^ blockade was reduced in neurons transfected with GluN2A^N615K^ and that this effect was more pronounced in the presence of ifenprodil, when a higher proportion of activated receptors contain GluN2A subunits. Third, we assessed the impact of GluN2A^N615K^ on current density (Fig. 4 *A*–*C* and *F*). We found that current density was reduced in neurons transfected with GluN2A^N615K^ and this effect was again more pronounced in the presence of ifenprodil. This reduction in current density is consistent with the reduction in conductance we observed previously (Fig. 3). Taken together, these results show that, when the GluN2A^N615K^ mutation is expressed in cells which endogenously express NMDA receptors, it continues to have a profound influence on Mg^2+^ blockade and on current density.

## Discussion

In the present study, we investigated the functional consequences of GluN2A^N615K^, a heterozygous missense variant found to have arisen *de novo* in two unrelated people with early onset epileptic encephalopathy. Using heterologous systems, we showed that the GluN2A^N615K^ variant results in major alterations to physiologically crucial aspects of NMDA receptor function: an almost complete loss of Mg^2+^ blockade and a four‐fold reduction in conductance. Importantly, the variant continues to markedly reduce Mg^2+^ blockade and conductance when expressed in NMDA receptor triheteromers with one wild‐type GluN2 subunit, and also continues to have effect when expressed in cortical neurons. These findings strengthen the evidence that the GluN2A^N615K^ variant causes the severe neurodevelopmental disorder experienced by its carriers.

### GluN2A^N615K^ reduces Mg^2+^ blockade

The marked reduction in Mg^2+^ blockade that we observed with the GluN2A^N615K^ mutation is in keeping with previous work identifying the affected residue as important for Mg^2+^ blockade (Wollmuth *et al*. 1998) and with the disruptive replacement of an asparagine with a positively‐charged lysine. Our finding is also consistent with previous work reporting minimal Mg^2+^ blockade in GluN2A^N615K^ diheteromers (Endele *et al*. 2010). We additionally demonstrated a reduction in Mg^2+^ blockade when GluN2A^N615K^ is expressed in primary neurons. The smaller magnitude of reduction in Mg^2+^ blockade seen in neurons probably reflects the presence of endogenous GluN2B subunits. We directly addressed the impact of GluN2A^N615K^ when expressed as part of triheteromeric NMDA receptors with wild‐type partner subunits and found that Mg^2+^ blockade was reduced to an intermediate extent. This is an important finding because disease‐associated mutations in *GRIN2A* have so far only been found heterozygously. Observing an effect of the variant despite the presence of wild‐type subunits further supports a role for GluN2A^N615K^ in disease causation. This finding indirectly supports the disease relevance of other NMDA receptor pore mutations where functional consequences have been established in diheteromers (Fedele *et al*. 2018; Fernández‐Marmiesse *et al*. 2018).

Interestingly, we observed an impact of partner subunit identity on Mg^2+^ blockade in GluN2A^N615K^‐containing triheteromers: receptors with a wild‐type GluN2A partner subunit showed higher Mg^2+^ blockade than those with a wild‐type GluN2B partner subunit. However, we did not see an impact of partner subunit identity on single‐channel conductance. The mechanism of this intriguing finding and the extent to which it applies to other receptor properties requires additional study.

A reduction in Mg^2+^ blockade would be hypothesized to have substantial impact on neuronal physiology and synaptic plasticity because voltage‐dependent Mg^2+^ blockade is essential to the role of the NMDA receptor as a molecular coincidence detector (Huganir & Nicoll, 2013). Alterations to this property could therefore be anticipated to result in deficits in learning and memory, as may be reflected in the severe and profound intellectual disability of carriers. However, plasticity is dependent on many interacting factors: additional receptor properties such as calcium permeability, expression of other NMDA receptor subunits and expression of other receptor types. Furthermore, deficits in plasticity caused by reduced or increased expression of NMDA receptor subunits have been associated with behavioural consequences in several (Sakimura *et al*. 1995; Kiyama *et al*. 1998; Roberts *et al*. 2009) but not all studies (Okabe *et al*. 1998). Further studies are needed to assess the potential impact of the GluN2A^N615K^ variant on synaptic plasticity, which would be aided by a transgenic animal model.

### GluN2A^N615K^ reduces conductance

We found that the GluN2A^N615K^ variant reduced single‐channel conductance by around four‐fold when expressed as diheteromers. Triheteromers containing only one GluN2A^N615K^ subunit showed two conductances: one similar to GluN2A^N615K^ diheteromers and one intermediate conductance equivalent to around two‐thirds of wild‐type. We also found that NMDA‐evoked current density was reduced in neurons expressing GluN2A^N615K^, suggesting that any acute compensation by other receptor subunits was insufficient to overcome the reduction in conductance associated with the GluN2A^N615K^ variant. Our finding of reduced conductance adds to previous work reporting similarly marked reductions in conductance following mutations of equivalent residues neighbouring the location of the GluN2A^N615K^ variant in GluN1 and GluN2B subunits (Behe *et al*. 1995; Premkumar *et al*. 1997; Schneggenburger & Ascher, 1997). In addition to this probable direct impact of the altered residue on ion permeation through the narrow internal region of the pore (Song *et al*. 2018), it is possible that other charged regions that strongly influence ion permeation (e.g. the DRPEER motif in the GluN1 subunit) (Wollmuth, 2018) may also be indirectly affected by the GluN2A^N615K^ variant, contributing to effects on single‐channel conductance. Our finding that glutamate deactivation rates are unaffected by the mutation shows that the overall time course of receptor openings is similar. A reduction in conductance is of physiological importance because it implies that less charge is passed by activated NMDA receptors containing GluN2A^N615K^ subunits. This would reduce the ability of a receptor to contribute to neuronal excitability, and also potentially alter the receptor's ionotropic signaling pathways.

In summary, the present study has strengthened the evidence indicating that the disease‐associated variant GluN2A^N615K^ is associated with substantial changes in physiologically crucial properties of NMDA receptors. This information can be used to inform genetic counselling. Our work highlights NMDA receptor‐related synaptic transmission as probable candidates for disruption in the pathogenesis of neurodevelopmental disorders. Future work could usefully explore the impact of this mutation *in vivo* in synaptic plasticity at circuit and behavioural levels.

## Additional information

### Competing interests

The authors declare that they have no competing interests.

### Author contributions

KFMM, KBH, PAS, GEH and DJAW were responsible for the conception and design of the experiments. KFMM and KBH were responsible for the collection and assembly of data. KFMM, KBH, PAS, GEH and DJAW were responsible for the analysis and interpretation of data. KFMM, KBH, PAS, GEH and DJAW were responsible for drafting the article or revising it critically for important intellectual content. All authors approved the final version of the manuscript submitted for publication, and all persons designated as authors qualify for authorship and all those who qualify for authorship are listed.

### Funding

This work was funded by the Wellcome Trust (WT102838) and National Institutes of Health (GM103546 and NS097536).
